# Longevity of implantable cardioverter defibrillators: a comparison among manufacturers and over time

**DOI:** 10.1093/europace/euv296

**Published:** 2015-11-25

**Authors:** Simon von Gunten, Beat A. Schaer, Sing-Chien Yap, Tamas Szili-Torok, Michael Kühne, Christian Sticherling, Stefan Osswald, Dominic A.M.J. Theuns

**Affiliations:** 1 Department of Cardiology, University Hospital, Basel, Switzerland; 2 Department of Cardiology, Erasmus Medical Center, Rotterdam, The Netherlands

**Keywords:** Longevity, Implantable cardioverter-defibrillator, Survival, Cardiac resynchronization therapy

## Abstract

**Aims:**

Longevity of implantable cardioverter defibrillators (ICDs) is crucial for patients and healthcare systems as replacements impact on infection rates and cost-effectiveness. Aim was to determine longevity using very large databases of two teaching hospitals with a high number of replacements and a rather homogeneous distribution among manufacturers.

**Methods and results:**

The study population consists of all patients in whom an ICD was inserted in. All ICD manufacturers operating in Switzerland and the Netherlands and all implanted ICDs were included. Implantable cardioverter defibrillator replacements due to normal battery depletion were considered events, and other replacements were censored. Longevity was assessed depending on manufacturers, pacing mode, implant before/after 2006, and all parameters combined. We analysed data from 3436 patients in whom 4881 ICDs [44.2% VVI-ICDs, 27.4% DDD-ICDs, 26.3% cardiac resynchronization therapy (CRT)-ICDs, 2.0% subcutaneous ICDs] were implanted. The four major manufacturers had implant shares between 18.4 and 31.5%. Replacement due to battery depletion (27.4%) was performed for 1339 ICDs. Patient survival at 5 years was 80.1%. Longevity at 5 years improved in contemporary compared with elderly ICDs [63.9–80.6% across all ICDs, of 73.7–92.1% in VVIs, 58.2–76.1% in DDDs, and of 47.1–66.3% in CRT defibrillators, all *P* value < 0.05]. Remarkable differences were seen among manufacturers, and those with better performance in elderly ICDs were not those with better performance in contemporary ones.

**Conclusion:**

Implantable cardioverter defibrillator longevity increased in contemporary models independent of manufacturer and pacing mode. Still, significant differences exist among manufacturers. These results might impact on device selection.

**What's new?**

- Several studies on implantable cardioverter defibrillator (ICD) longevity have been published. Except one, all studies included <1300 ICDs and the number of replaced ICDs was low, thus compromising the significance of results. This one is a study with the largest number of ICDs included so far.
- It shows a rather homogenous distribution of ICDs from the four largest manufacturers (St. Jude Medical 31.5%, Biotronik 25.0%, Boston Scientific 19.4%, Medtronic 18.4%) compared with earlier studies with much more skewed distributions.
- Previous studies showed minor differences between manufacturers, while this study, especially in more contemporary ICDs, demonstrated huge differences. Longevity at 6 years in cardiac resynchronization therapy-ICDs, e.g. was 97.6% with the best manufacturer, compared with 46.3% with the second best.

## Introduction

In selected patients, implantable cardioverter defibrillators (ICDs) are considered standard of care for primary and secondary preventions of sudden cardiac death.^1–5^ Devices reduce morbidity and mortality due to efficient termination of ventricular tachyarrhythmias. Yet undesired factors such as complications during and early after implantation,^3^ deterioration in quality of life,^4,6^ lead failure,^1^ and advisories^2^ are increasingly identified. The most disturbing element for patients, apart from repeated inappropriate shocks,^7^ however, is the necessity of a surgical procedure for ICD replacement due to premature or even timely battery depletion. Besides the cost of the procedure, there is the risk of periprocedural complications such as damage to the leads, bleeding, and infections.^8,9^

During the past decade, several studies on ICD longevity have been published.^10–20^ With the exception of one large series,^18^ all studies included <1300 ICDs and the number of actually replaced ICDs was low, thus compromising the significance of the results. In addition, analyses were performed only among different manufacturers, not accounting for temporal trends in longevity between different models. Overall, it was confirmed that single-chamber defibrillators (VVI-ICDs) have better longevity than dual-chamber defibrillators (DDD-ICDs) and the latter a better one than cardiac resynchronization therapy defibrillators (CRT-Ds).

In contrast, results regarding longevity advantages of one manufacturer as against another were heterogeneous and sometimes even contradictory. Nevertheless, positive results have been used by some manufacturers for advertising campaigns in major electrophysiological journals.

The aim of this study was to perform a comprehensive analysis of longevity in a large series of ICDs not only with respect to different manufacturers, but also to different device models, pacing mode and to determine temporal trends of ICD longevity.

## Methods

The study population consists of all consecutive patients in whom an ICD was implanted at the Erasmus Medical Center, Rotterdam, the Netherlands and at the University of Basel Hospital, Basel, Switzerland. The implant period encompasses from March 1994 to January 2014. Some of these patients and ICDs have already been included in two previous studies.^14,15^ As this study is an analysis of *all* ICD implantations in both hospitals, we still considered these data for the present study.

Baseline characteristics of the patients and details of follow-up visits were recorded prospectively, as well as data regarding implanted ICD, such as manufacturer, device model, and pacing mode (single chamber, dual chamber, CRT). Date of last access to the database was 31 May 2014 for patients at the Erasmus Medical Center, Rotterdam and 30 June 2014 for patients at the University of Basel Hospital.

**Figure 1:**
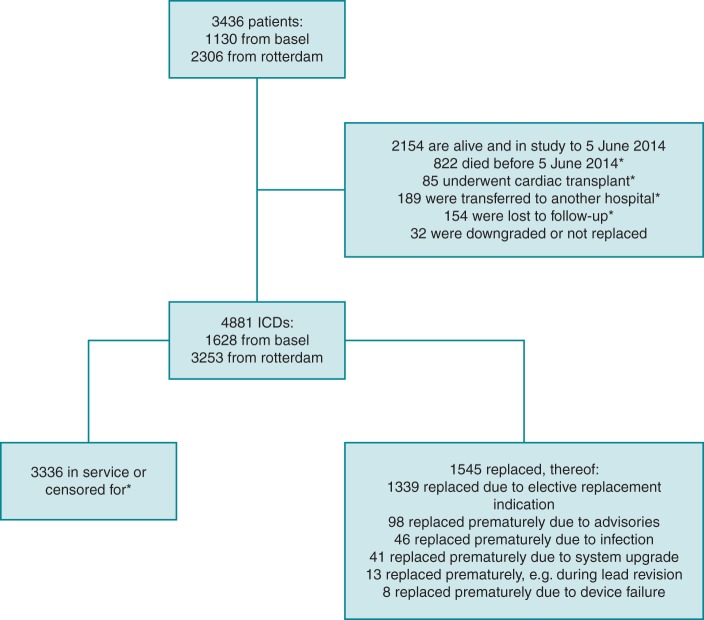
Flow chart of patients and ICDs.

ICD replacements due to normal battery depletion, a so-called elective replacement indication (ERI), were considered events. Service time in months was calculated as the difference between implant date and replacement date or between replacement date and replacement date, as appropriate. In all other replacement settings such as upgrade, advisory, or removal for infection, the device was censored at the date of the procedure. Implantable cardioverter defibrillators still in service were censored at the date of last database access. Implantable cardioverter defibrillators still in service but lost to follow-up were censored at the date of last follow-up. Implantable cardioverter defibrillators of patients who died or were transplanted were censored at the date of these corresponding events. *Figure 1* displays a flow chart of both patients and ICDs.

All manufacturers of ICDs ever operating in the two countries and all their ICDs used in the two hospitals were included. These were in alphabetical order: Biotronik (Berlin, Germany), Boston Scientific (earlier Guidant; Marlborough, MA), Cameron Health (San Clemente, CA), Intermedics (formerly in Le Locle, Switzerland), Medtronic (Minneapolis, MN), St. Jude Medical (St. Paul, MN), and Sorin (earlier ELA, Milano, Italy).

For the purpose of this article, the term longevity is used for device longevity and the term survival for patient survival.

The first analysis was performed across all ICDs of a given manufacturer, and longevity was compared. The second analysis was performed across different pacing modes, wherefore all manufacturers were merged and longevity was compared between pacing modes. The third analysis was performed across ICDs according to pacing mode in between individual manufacturers. For the fourth analysis, a ranking of longevity at the time points 4 and 5 years was performed and a corresponding chart of the best five and worst three devices was made. An analysis across ICDs with a similar production year grouped together was also performed here. Finally, a comparison of longevity across manufacturers according to pacing mode of ICDs implanted before 2006 and thereafter was performed. This time point was chosen as it was close to the median of all devices. No individual analyses were performed in those devices with <20 implants.

In both hospitals, an institutional review board approval was not required as the study was designed as an analysis of retrospective registry data. Patient information was de-identified in the merged Excel file that was used for all analyses.

Kaplan–Meier method was used to determine longevity, and differences were compared with log-rank testing. A *P* value of <0.05 was considered as statistically significant. All statistical analyses were performed using IBM SPSS software, version 22.

## Results

We analysed data from 3436 patients [median age 62 years (IQR 52–70)] in whom 4881 ICDs have been implanted. Important baseline characteristics of the patients are shown in *Table 1*. During a follow-up of median 53 months (IQR 24–87), 822 (23.9%) patients died. This results in a 4-, 5-, and 10-year cumulative survival of 83.3, 80.1, and 59.1%, respectively. The corresponding Kaplan–Meier curve (Supplementary material online, *Figure S1*) indicates a linear mortality rate. Appropriate ICD therapy was delivered to 972 patients (28.3%).

**Table 1:** Baseline characteristics of the 3436 patients

Male gender	2721 (79%)
Age (mean ± SD)	59 ± 14
Follow-up (mean ± SD)	60.3 ± 44.4
Ejection fraction (mean ± SD)	32 ± 13
QRS width (mean ± SD)	127 ± 35
Primary prevention	1975 (57%)
Ischaemic cardiomyopathy	2525 (74%)
Myocardial infarction	1825 (53%)
Percutaneous coronary intervention	1069 (31%)
Coronary artery bypass	769 (22%)
Diabetes mellitus	694 (20%)
Creatinine level (μmol/L, mean ± SD)	103 ± 69
NYHA Class I	792 (23%)
NYHA Class II	1680 (49%)
NYHA Class III	907 (27%)
NYHA Class IV	39 (1%)
ACE/ARB therapy	2640 (77%)
Beta-blocker therapy	2621 (76%)
Diuretic therapy	2029 (59%)
Statin therapy	1978 (58%)
Amiodarone therapy	685 (20%)
Digoxin therapy	461 (13%)

SD, standard deviation; ACE, angiotensin converting enzyme inhibitor; ARB, angiotensin receptor blocker.

Overall, 2158 (44.2%) VVI-ICDs, 1340 (27.4%) DDD-ICDs, 1284 (26.3%) CRT-ICDs, and 99 (2.0%) subcutaneous ICDs were inserted. In *Table 2*, details on manufacturers and pacing modes are presented. The percentages of ICDs used from the different manufacturers in a downward frequency are as follows: St. Jude Medical 31.5%, Biotronik 25.0%, Boston Scientific 19.4%, Medtronic 18.4%, Sorin ELA 3.2%, Cameron Health 2.0%, and Intermedics 0.4%. In the Supplementary material online, *Table S1*, all ICD models are displayed in detail. As shown in *Figure 1*, 1339 ICDs were replaced for ERI (27.4%, range 1–5 per patient) and 206 (4.6%) for other reasons.

**Table 2:** Manufacturers, ICD, pacing modes (upper row) and replaced ICDs (lower row), shown in alphabetical order

Manufacturer	VVI	DDD	CRT	Total	% Replaced
Biotronik	645	346	228	1219	
	149	126	23	298	24.5%
Boston Scientific	413	275	259	947	
	118	113	80	311	31.8%
Intermedics	21	0	0	21	
	19	0	0	19	90.5%
Medtronic	449	182	267	898	
	181	78	97	356	39.6%
St. Jude Medical	625	388	526	1539	
	94	85	116	295	19.2%
Sorin ELA	10	144	4	158	
	1	48	0	49	31.0%
Total					
Devices	2158	1340	1284	4782	
Replaced	557	455	316	1328	
% Replaced	25.8%	34.0%	24.6%	27.8%	
Cameron Health	99			99	
	11			11	11.1%

ICD, implantable cardioverter defibrillator; VVI, single-chamber ICD; DDD, dual-chamber ICD; CRT, cardiac resynchronization therapy ICD.

**Figure 2:**
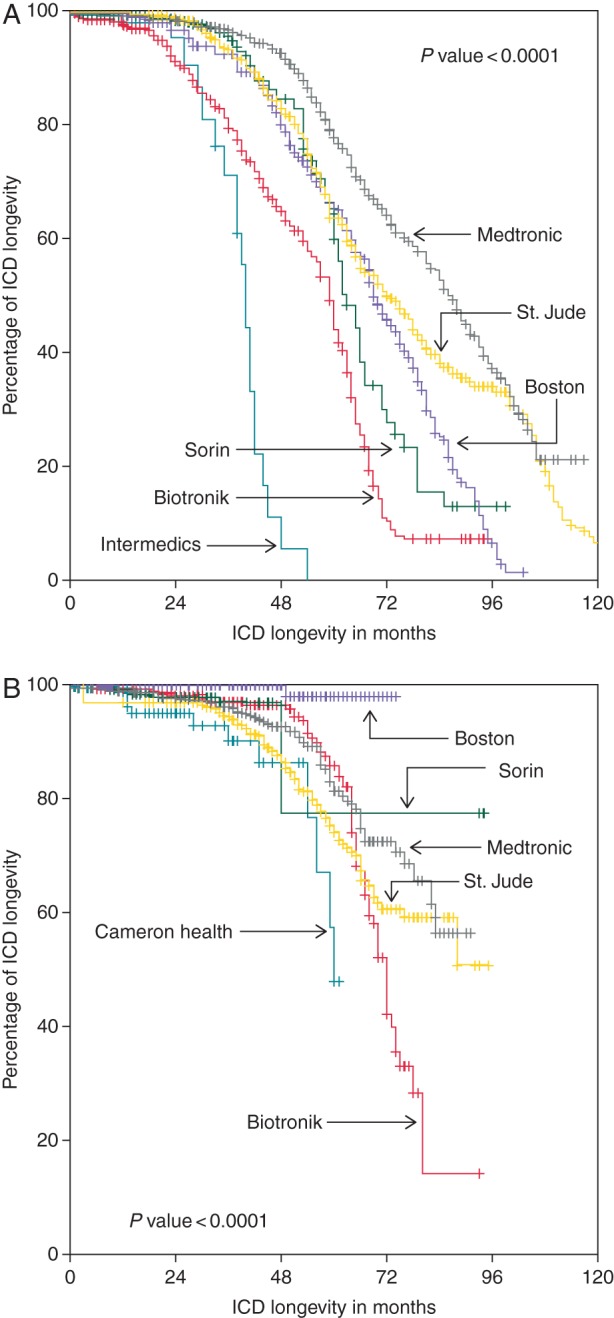
Comparison of longevity according to manufacturers (total *n* = 4881, before 2006 *n* = 2198, after 2006 *n* = 2683).

**Figure 3:**
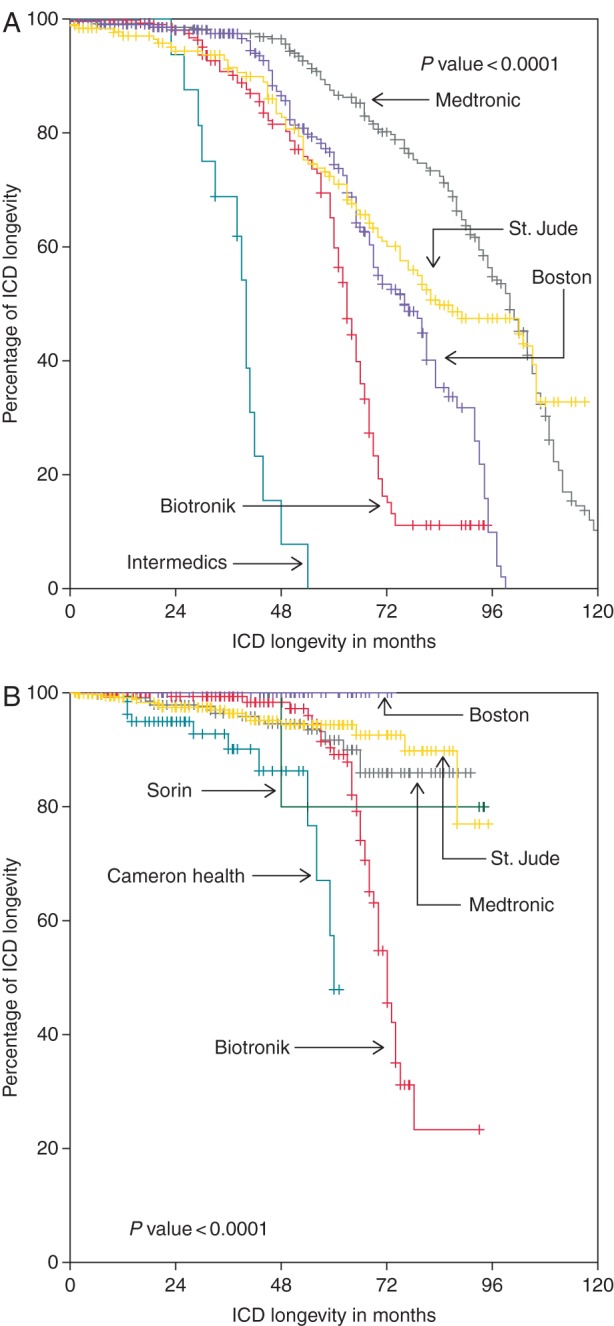
Comparison of longevity of VVI-ICDs manufacturers (total *n* = 2158, before 2006 *n* = 985, after 2006 *n* = 1272).

**Figure 4:**
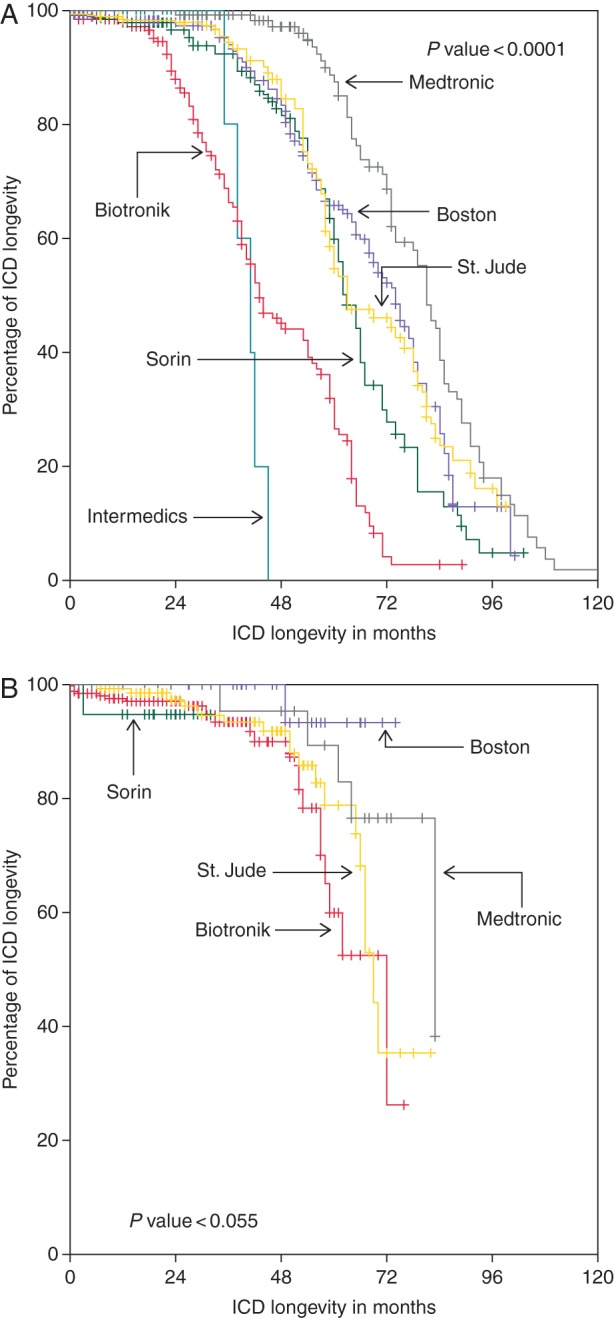
Comparison of longevity of DDD-ICDs manufacturers (total *n* = 1340, before 2006 *n* = 816, after 2006 *n* = 524).

**Figure 5:**
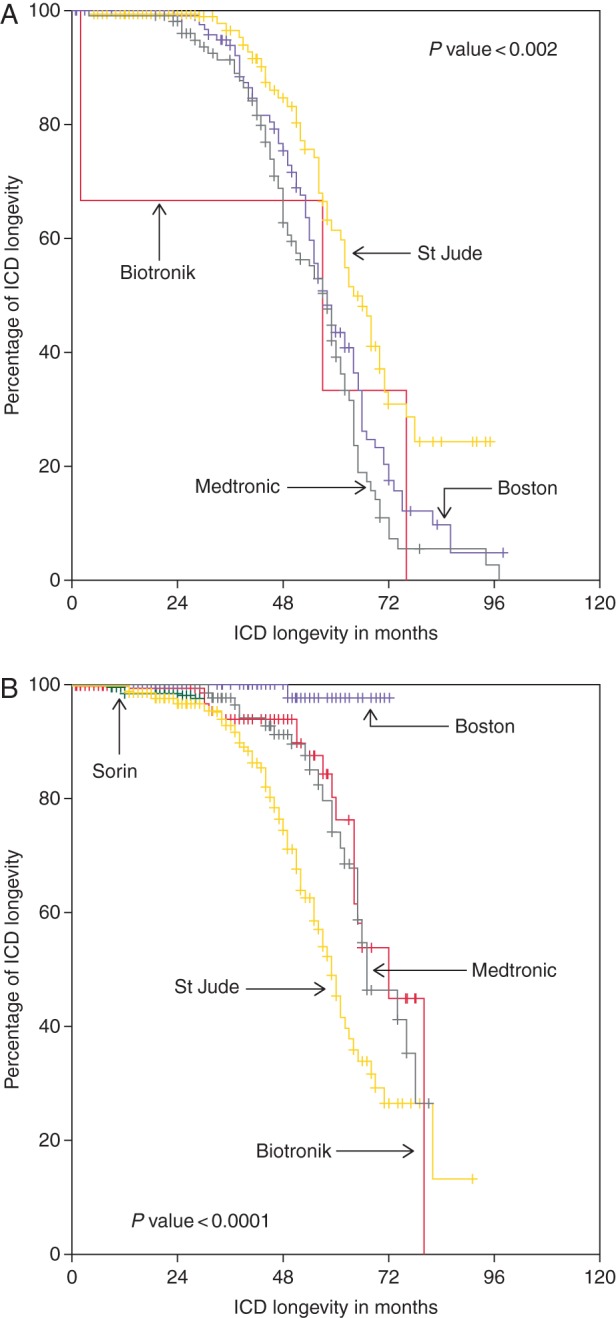
Comparison of longevity of CRT-ICDs (total *n* = 1284, before 2006 *n* = 397, after 2006 *n* = 887).

Overall longevity, is shown in the Supplementary material online, *Figure S2*. It was 85.1% at 4 years and 69.8% at 5 years. Longevity before 2006 (2198 ICDs, 45%) and thereafter (2683 ICDs, 55%) was compared. These overall values at 5 and 6 years for VVI-ICDs, DDD-ICDs, and CRT-Ds were 80.1 and 62.7, 62.0 and 43.4, and 56.3 and 27.4%, respectively. The difference according to pacing modes before and after 2006 is depicted in the Supplementary material online, *Figure S3*. *Figures 2–5* show the comparisons of longevity according to manufacturers in the two implant periods before and after 2006, overall as well in the different pacing modes.

Overall, cumulative longevity markedly improved from 63.9 to 80.6% at 5 years and from 44.9 to 61.6% at 6 years (*P* ≤ 0.001). The same holds true for different pacing modes (all *P≤*0.001). On the level of manufacturers, intra- as well as intermanufactural improvement was diverse and occasionally even remarkable. Whereas in the early period, usually Medtronic ICDs had the best longevity, this changed to Boston Scientific ICDs in contemporary devices. Boston Scientific ICDs exhibited an excellent, sometimes even 100% longevity, independent of pacing mode (see *Table 3* for more details).

**Table 3:** Comparison of longevity of devices implanted until December 2005 and thereafter (highlighted is the best performance in the corresponding group) according to the manufacturer and pacing mode

	Before 2006		Thereafter	
	5-year longevity (%)	6-year longevity (%)	5-year longevity (%)	6-year longevity (%)
All ICD models				
All manufacturers**	63.9	44.9	80.6	61.6
Biotronik**	44.0	10.5	81.4	42.1
Boston**	65.1	45.7	**98.0**	**98.0**
Medtronic^||^	**77.7**	**64.1**	85.8	72.6
St. Jude Medical**	64.3	49.8	74.1	60.7
Sorin^||^	59.8	27.8	77.5	77.5
Intermedics	0	0	n.a.	n.a.
Cameron Health	n.a.	n.a.	47.9	n.a.
VVI				
All manufacturers**	73.7	56.4	92.1	76.0
Biotronik**	59.8	15.2	89.1	45.6
Boston**	74.3	53.3	**100.0**	**100.0**
Medtronic^||^	**86.7**	**80.1**	91.7	85.9
St. Jude Medical**	70.9	60.1	94.3	92.6
Sorin	n.a.	n.a.	80.0	80.0
Intermedics	0	0	n.a.	n.a.
Cameron Health	n.a.	n.a.	47.9	n.a.
DDD				
All manufacturers**	58.2	40.8	76.1	50.9
Biotronik**	26.6	4.2	60.0	26.3
Boston^#^	65.8	52.2	**93.3**	**93.3**
Medtronic^||^	**87.5**	**68.6**	89.3	76.5
St. Jude Medical^||^	54.7	46.0	78.7	35.3
Sorin^||^	59.8	27.8	n.a.	n.a.
CRT				
All manufacturers**	47.1	21.2	66.3	43.0
Biotronik**	0	0	76.2	44.9
Boston**	43.5	17.5	**97.6**	**97.6**
Medtronic**	39.2	7.4	74.1	46.3
St. Jude Medical^||^	**61.5**	**30.9**	45.3	26.5

n.a., not applicable, i.e. not manufactured in this period, not implanted in the two hospitals, or time point not reached; ICD, implantable cardioverter defibrillator; VVI, single-chamber ICD; DDD, dual-chamber ICD; CRT, cardiac resynchronization therapy ICD.

^||^ *P* = n.s., ^#^*P* ≤ 0.05, **P ≤ 0.001.

The SSupplementary Data, presents the charts of the five longest lasting and the three shortest lasting devices at the time points of 4 and 5 years, according to pacing mode. Medtronic held 10/30 positions in the longest lasting charts, Biotronik and Boston Scientific both 8/18 in the shortest lasting charts. The Supplementary material online, *Tables S3* and *S4*, depicts longevity of ICD with a similar year of production and a comprehensive 5-year longevity of all ICD models.

Comparing patient survival with ICD longevity, the ICDs always performed poorer. In VVI, 5-year survival and longevity were 85.1% in patients and 80.1% in ICDs, in DDD 77.0 and 62.0%, and in CRT 72.8 and 56.3%, respectively.

## Discussion

This article represents the largest study on ICD longevity so far and includes all major manufacturers in a reasonably balanced proportion. Although all manufacturers have improved on longevity of their devices, large differences in longevity still exist. Before 2006, Medtronic VVI-ICDs, Medtronic DDD-ICDs, and St. Jude Medical CRT-Ds exhibited the best longevity. In the current era (i.e. after 2006), however, Boston Scientific ICDs outperformed all other manufacturers with regard to longevity. In spite of these partly remarkable improvements, survival of patients is better than ICD longevity and not vice versa as should be the case.

### Comparison with other studies regarding differences among manufacturers

Of the nine studies on overall longevity, only three provided reliable data in a larger group of patients and devices.^13,15,18^ All cover an extensive implant period of 13–21 years and therefore report on a vast range of ICD models. Superior longevity of VVI pacing mode (median longevity 6–7.3 years) over DDD pacing mode (5–5.7 years) and over CRT pacing mode (median longevity 4.2–5.2 years) is consistently reported, and these differences can be explained primarily by the increasing percentage of pacing. Main analyses were carried out by lumping together all ICDs by a given manufacturer independently of pacing mode and model. Medtronic excelled throughout with median longevities from 5.8 to 7.6 years. Runners-up were Boston Scientific and St. Jude Medical (5–5.4 and 5–5.8 years, respectively), with Biotronik being at the tail end (4.6–4.7 years). Sorin ICDs were incorporated in only one study. Even though these studies are not without their merits, one of their major limitations lies in the very skewed range of implant shares of the different manufacturers [6–61% in (10), 6–49% in (15), 18–49% in (12)]. In the present study, with the largest numbers of ICDs implanted, this range was much smaller (20–34%), which makes this analysis more robust.

As one can appreciate by looking at data of longevity analysed either by lumping together all ICDs of different manufacturers or by comparing manufacturers in a given pacing mode independent of time, they often do not reflect the whole truth. In our study, for example, Kaplan–Meier analysis suggests that Biotronik CRT-Ds have by far the best performance, but the analysis with regard to temporal trends gives another picture. Here, Biotronik is in the range of Medtronic and by far behind Boston Scientific. The poor performance of most Medtronic and Boston Scientific CRT-Ds in the first half of the study period leads to this distorted result.

### Temporal trends

The comparison of *longevity at 6 years* of ICDs implanted before and after 2006 shows first and foremost an absolute increase in longevity of 10% (DDD) and 20% (VVI & CRT), yet differences between manufacturers exist regarding absolute values. In contemporary VVI-ICDs, Boston Scientific excelled with 100% longevity at 6 years, Biotronik was subprime with <50% longevity, and the other manufacturers were satisfactory. In DDD pacing mode, Biotronik and St. Jude Medical were subprime, Sorin and Medtronic moderate, and again Boston Scientific excelled. The most remarkable difference was seen in CRT-Ds, with Boston Scientific again showing an almost 100% longevity at 6 years compared with all other manufacturers with St. Jude Medical being at the tail end with only 26% longevity. This poor performance seems at first glance contradictory to data from the study of Alam *et al.*^10^ in which St. Jude Medical longevity at 4 years was reported as 92%. At second glance, these results are hampered as they depend only on 57 implanted CRTs and actually four replacements.

Temporal trends have been analysed with a cut-off in the year 2002.^13,15,18^ In two studies,^13,15^ no significant improvement was observable. In one study,^18^ mean longevity of replaced devices improved by 12 months in DDD and CRT and by 20 months in VVI pacing mode. The value of these results is altogether hampered by a severe dysbalance between groups (only 12% of ICDs were implanted before 2002).

The difference among manufacturers in the second half of the study period and the striking improvement in longevity in all Boston Scientific ICD pacing modes might be due to their specific battery technology. Boston Scientific incorporates a Lithium Manganese battery with a capacity of almost 2 ampere-hours in their contemporary devices, whereas other manufacturers usually use Lithium Silver Vanadium with less ampere-hours. Further studies are needed to confirm or repudiate this hypothesis.

### Longevity and survival

Only one paper described the interrelation of longevity and survival.^12^ In 2005, Hauser reported a 4-year survival of 79% and refers to unpublished data with 4-year longevity of 54%. While survival is pretty similar to the present study (here 83.3%), a huge difference exists regarding longevity (here 85.1%). This can be explained, at least in part, by the temporal trend of increased longevity. However, it is intriguing that also in newer ICDs, 5-year survival is still better than 5-year longevity. While the difference was only 5% in VVI-ICDs (and thus acceptable), it rose to 15% in DDD-ICDs. In CRT-ICDs implanted in an admittedly high-risk population with a higher mortality, the difference was 23%. This indicates that a better battery technology is foremost required for this group of patients.

### Impact of the number of shocks, pacing percentage, and pacing thresholds

We could not perform special analyses with regard to these possible confounders as they were available only in a minority of devices. Previous studies^15,18^ negated a significant influence of shocks on longevity. Pacing percentage and thresholds have been shown to be different in between manufacturers, but only when all ICDs were grouped together and not in different pacing modes.^15^ Predictors of decreased longevity were the LV output per se,^10^ an increase in overall pacing output of 1 V,^18^ and a 10% increase in pacing percentage,^13,18^ but no analyses were performed to show that they were different among manufacturers. A limitation of these analyses is the fact that only values determined at last follow-up were considered, thus giving a skewed picture of reality with the use of a random point in time. In addition, all these possible confounders probably are smoothed by the large number of devices included in the present study.

### Limitations

This study has the inherent limitations of all registry data. An attribution bias (the physician could implant a presumed long-lasting ICD in a young patient and vice versa) might be present but is limited by the two-centre design of the study and consequently different perceptions and daily practice. Implantable cardioverter defibrillators were replaced at ERI and longevity calculated according to it and not to the end of life of the battery, thus reducing the real longevity. However, this was done in all studies on longevity so far. As all ICDs implanted in both centres were included over a long period of time, some results do not reflect longevity of currently available and implanted ICDs. However, this is overcome in part by the separate analysis on temporal trends according to the median time of implant. If one wants to have data on longevity of rather long-lasting devices, requesting results that reflect longevity of ICDs implanted in the past few years is an obvious oxymoron.

## Conclusion

At 5 years, overall ICD longevity is 70% and thus lower than the patient survival of 80%. A highly significant improvement of longevity was seen in more contemporary models implanted after 2006. However, this improvement was unequal among the manufacturers. Results might impact on device selection.

## Supplementary material

Supplementary material is available at *Europace* online.

## Supplementary Material

Supplementary Data
