# Supplementary material for: Longevity of implantable cardioverter defibrillators: a comparison among manufacturers and over time
Source: Europace. 2015 Nov 25;18(5):710–7. doi: 10.1093/europace/euv296 (PMC4880113; doi:10.1093/europace/euv296)
Supplement: Supplementary Data [file euv296_supplementary_data.zip › euv296supp_tables4.docx]

Supplemental table 4:

Longevity of devices (at least 20 implants and 3 replacements) at the time point 5 years, ranked according to falling percentage

year implanted/replaced 5 year longevity

VVI

St. Jude Medical: Atlas II VR V-168 VVI 2006 105 4 97.5

Medtronic: Marquis 7230 VR VVI 2004 50 26 97.0

St. Jude Medical: Current RF 1207 VVI 2007 84 4 95.7

Biotronik: Lumax 540 VR-T VVI 2007 259 8 95.3

Boston Scientific: Vitality 2 EL T177 VVI 2004 48 10 94.5
Medtronic: Entrust VVI 2004 32 6 93.3

Medtronic: GEM 7227 VVI 1999 56 44 92.1

St. Jude Medical: Atlas VR V-193 VVI 2003 103 17 91.5

Boston Scientific: Vitality 2 T175 VVI 2004 142 21 90.2

Medtronic: Virtuoso D154VWC VR VVI 2007 77 10 90.2

Biotronik: Lumax 300 VR-T VVI 2007 67 24 87.5

Medtronic: GEM III 7231 VVI 1999 88 58 86.4

Boston Scientific: Ventak Prizm 2 1860 VVI 2000 35 26 83.9

Biotronik: Lumax 340 VR-T VVI 2007 42 15 81.4

Boston Scientific: Ventak Mini 4 1793 VVI 2000 34 22 79.5
St. Jude Medical: Atlas VR V-199 VVI 2001 72 36 73.2

Biotronik: Lexos VR-T VVI 2003 56 31 67.6

Biotronik: Lumos VR-T VVI 2005 82 44 60.0
Biotronik: Belos VR-T VVI 2003 22 14 36.7

Boston Scientific: Ventak Prizm HE 1852 VVI 2001 28 23 0.0

St. Jude Medical: Profile 186 VVI 1999 20 12 0.0

DDD

St. Jude Medical: Atlas DR V-243 DDD 2003 62 21 94.7
St. Jude Medical: Current RF 2207 DDD 2007 47 3 92.7
Medtronic: GEM II DR 7271 DDD 1999 32 19 91.3
Medtronic: Virtuoso D154AWG DR DDD 2007 26 5 89.3

Boston Scientific: Ventak Prizm 2 1861 DDD 2000 46 29 83.1
Boston Scientific: Vitality 2 T165 DDD 2004 77 20 83.1

Biotronik: Lumax 540 DR-T DDD 2007 106 9 80.9

Medtronic: Marquis 7274 DR DDD 2004 39 17 76.2

St. Jude Medical: Atlas II DR V-268 DDD 2006 59 15 75.6

Biotronik: Lumos DR-T DDD 2005 28 11 73.4

Sorin ELA: Ovatio DR-6550 DDD 2005 56 26 69.0

Biotronik: Lexos DR-T DDD 2003 25 18 63.8

Boston Scientific: Vitality 1871 DDD 2004 65 38 61.3

Sorin ELA: Alto DR 614 DDD 2001 28 13 23.8
St. Jude Medical: Atlas DR V-240 DDD 2001 59 41 19.4
Biotronik: Tachos DR DDD 2003 44 10 11.2

Boston Scientific: Ventak Prizm HE 1853 DDD 2000 22 15 9.4

Biotronik: Tachos Atx DDD 2003 42 26 8.1
Biotronik: Phylax AV DDD 2000 49 41 0.0

CRT

Biotronik: Lumax 540 HF-T CRT 2007 148 9 89.2

Medtronic: InSync 7272 CRT 2002 25 9 77.8
St. Jude Medical: Promote RF 3213 CRT 2009 104 10 77.3
Boston Scientific: Contak Renewal 4 RF H230 CRT 2005 31 10 73.7
Medtronic: Concerto C 174 CRT 2006 52 23 72.4
St. Jude Medical: Atlas HF V-341 CRT 2004 100 38 68.3

Biotronik: Lumax 340 HF-T CRT 2007 30 11 61.4

Boston Scientific: Contak Ren. 4 AVT M177 HE CRT 2005 23 13 49.5
Medtronic: InSync Sentry 7298 CRT 2005 46 34 47.8

St. Jude Medical: Atlas II+ HF V-367 CRT 2007 143 56 37.4

Medtronic: InSync III Marquis 7279 CRT 2005 47 28 9.5
Boston Scientific: Contak Renewal H135 CRT 2002 21 15 0.0

Boston Scientific: Contak Renewal 2 H155 CRT 2002 30 16 0.0

Medtronic: Consulta D234TRK CRT 2008 23 3 0.0
