# Supplementary material for: Longevity of implantable cardioverter defibrillators: a comparison among manufacturers and over time
Source: Europace. 2015 Nov 25;18(5):710–7. doi: 10.1093/europace/euv296 (PMC4880113; doi:10.1093/europace/euv296)
Supplement: Supplementary Data [file euv296_supplementary_data.zip › euv296supp_tables1-3.pdf]

Supplemental table 1:

Overview of all implanted ICDs, in alphabetical order

Manufacturer	Model	implanted	replaced
Biotronik		1’219	298
	Belos VR-T	22	14
	Ilesto 7 CRT	1	0
	Ilesto 7 VRT	19	0
	Kronos LV-T	3	3
	Lexos A+/T	16	13
	Lexos DR-T	25	18
	Lexos VR-T	56	31
	Lumax 300 DR-T	11	4
	Lumax 300 VR-T	67	24
	Lumax 340 DR-T	14	5
	Lumax 340 HF-T	30	11
	Lumax 340 VR-T	42	15
	Lumax 540 DR-T	106	9
	Lumax 540 HF-T	148	9
	Lumax 540 VR-T	259	8
	Lumax 740 DR-T	24	0
	Lumax 740 HF-T	46	0
	Lumax 740 VR-T	82	0
	Lumos DR-T	28	11
	Lumos VR-T	82	44

	Phylax AV	49	41
	Tachos Atx	42	26
	Tachos DR	44	10
	Xelos DR-T	3	2
Boston Scientific		947	311
	Cognis CRT	76	1
	Contak CD 1823	11	4
	Contak Renewal 2 H155	30	16
	Contak Renewal 4 190	1	1
	Contak Renewal 4 AVT M177 HE	23	13
	Contak Renewal 4 H195	15	8
	Contak Renewal 4 HE H199	14	10
	Contak Renewal 4 RF H230	31	10
	Contak Renewal 4 RF H235	9	2
	Contak Renewal H135	21	15
	Energen CRT	24	0
	Energen DR	9	0
	Energen VR	28	0
	Incepta F 162	2	0
	Incepta P 163	1	0
	Incepta P 165	1	0
	Teligen CRT	2	0
	Teligen DR	28	0
	Teligen VR	82	0
	Ventak Mini 4 1793	34	22
	Ventak Prizm 1850	4	4
	Ventak Prizm 2 1851	9	7

	Ventak Prizm 2 1860	35	26
	Ventak Prizm 2 1861	46	29
	Ventak Prizm AVT 1900	5	3
	Ventak Prizm HE 1852	28	23
	Ventak Prizm HE 1853	22	15
	Vitality 1871	65	38
	Vitality 2 EL 167	9	0
	Vitality 2 EL T177	48	10
	Vitality 2 T165	77	20
	Vitality 2 T175	142	21
	Vitality A 155	3	1
	Vitality VR 1870	12	12
Cameron Health		99	11
	1010 SQ-RX	99	11
Intermedics		21	19
	Micron	7	7
	ResQmicron	14	12
Medtronic		898	356
	Concerto C 174	52	23
	Consulta D234TRK	23	3
	Entrust VVI	32	6
	Entrust DDD	4	2
	Evera	1	0

Evera XT DR	1	0
GEM 7227	56	44
GEM II 7271	1	0
GEM II DR 7271	32	19
GEM II DR 7273	1	1
GEM II VR 7229	4	4
GEM III 7231	88	58
GEM III 7275	15	9
GEM III 7276 AT	9	6
InSync 7272	25	9
InSync III Marquis 7279	47	28
InSync Sentry 7298	46	34
Intrinsic 7288	17	9
Jewel 7219	9	9
Jewel 7220	4	3
Jewel 7221	1	1
Jewel AF 7250 H	19	10
Marquis 7230 VR	50	26
Marquis 7274 DR	39	17
Maximo 7232	18	8
Maximo 7278	5	0
Microjewel 7221	8	7
Microjewel 7223	7	5
Protecta CRT	63	0
Protecta DDD	13	0
Protecta VVI	68	0
Secura VVI	26	0

	Secura DDD	1	0
	Virtuoso D154AWG DR	26	5
	Virtuoso D154VWC VR	77	10
	Viva-Q VT	10	0
St. Jude Medical		1’539	295
	Atlas DR V-240	59	41
	Atlas DR V-243	62	21
	Atlas HF V-341	100	38
	Atlas II DR V-268	59	15
	Atlas II VR V-168	105	4
	Atlas II+ HF V-367	143	56
	Atlas V-366	1	0
	Atlas VR V-193	103	17
	Atlas VR V-199	72	36
	Contour V 175	1	1
	Current RF 1207	84	4
	Current DR 2215	4	0
	Current VR 1215	5	0
	Current RF 2207	47	3
	Ellipse CD 1277	59	0
	Ellipse CD 1377	18	1
	Ellipse CD 2277	48	3
	Ellipse CD 2377	16	0
	Epic HF V-339	19	9
	Epic V-158	4	0
	Fortify CD 1233	138	7
	Fortify CD 2233	93	2

	Fortify CD 2259	1	0
	Photon micro 194	9	8
	Photon micro 232	1	1
	Photon V 194	1	0
	Photon V-230	3	2
	Profile 186	20	12
	Promote 3215	1	0
	Promote Quadra	1	0
	Promote RF 3213	104	10
	Unify Assura CD 3261	29	0
	Unify Assura CD 3361	6	0
	Unify CD 3235	1	0
	Unify CD 3251	2	0
	Unify CD 3235	118	3
	Ventritex 190	2	1
Sorin ELA		158	49
	Alto DR 614	28	13
	Alto DR 624	3	0
	Defender IV DR 612	15	8
	Ovatio DR-6550	56	26
	Ovatio VR-6250	5	1
	Paradym 8250	5	0
	Paradym 8550	14	0
	Paradym 8750	2	0
	Paradym 8758	1	0
	Paradym 9550	28	1
	Paradym SonR 9770	1	0

Supplemental table 2:
Longevity at 4 and 5 years of the 5 longest lasting and 3 shortest lasting ICD models (only analysed with at least 20 implants)
↑ indicates higher ranking, ↔ same ranking and ↓ lower ranking

ICD type	year	implants	replacements	% ICD longevity	
**VVI**					
At 4 years					
Mean value of all VVI-ICDs				90.1	
Medtronic GEM 7227	1999	56	44	100	
Medtronic Marquis 7230 VR	2004	50	26	100	
Boston Scientific Teligen	2008	82	0	100	
Boston Scientific Vitality 2 EL T177	2004	48	10	97.9	
St. Jude Medical Atlas II VR V-168	2006	105	4	97.5	
Biotronik Lumos VR-T	2005	82	44	74.6	
Biotronik Belos VR-T	2003	22	14	66.0	
Boston Scientific Ventak Prizm HE 1852	2001	28	23	45.8	
At 5 years					
Mean value of all VVI-ICDs				80.1	
St. Jude Medical Atlas II VR V-168	2006	105	4	97.5	↑
Medtronic Marquis 7230 VR	2004	50	26	97.0	↔
St. Jude Medical Current RF 1207	2007	84	4	95.7	↑
Biotronik Lumax 540 VR-T	2007	259	8	95.3	↑
Boston Scientific Vitality 2 EL T177	2004	48	10	94.5	↓
Biotronik Belos VR-T	2003	22	14	36.7	↑

Boston Scientific Ventak Prizm HE 1852	2001	28	23	0.0	↑
St. Jude Medical Profile 186	1999	20	12	0.0	↓
<u>DDD</u>					
At 4 years					
Mean value of all DDD-ICDs				80.4	
Biotronik Lumos DR-T	2005	28	11	100	
Boston Scientific Teligen	2008	28	0	100	
Medtronic GEM II DR 7271	1999	32	19	96.2	
Medtronic Virtuoso D154AWG DR	2007	26	5	95.2	
St. Jude Medical Atlas DR V-243	2003	62	21	94.7	
Biotronik Tachos Atx	2003	42	26	16.2	
Boston Scientific Ventak Prizm HE 1853	2000	22	15	9.4	
Biotronik Phylax AV	2000	49	41	5.7	
At 5 years					
Mean value of all DDD-ICDs				62.0	
St. Jude Medical Atlas DR V-243	2003	62	21	94.7	↑
St. Jude Medical Current RF 2207	2007	47	3	92.7	↑
Medtronic GEM II DR 7271	1999	32	19	91.3	↓
Medtronic Virtuoso D154AWG DR	2007	26	5	89.3	↓
Boston Scientific Ventak Prizm 2 1861	2000	46	29	83.1	↑
Boston Scientific Ventak Prizm HE 1853	2000	22	15	9.4	↑
Biotronik Tachos Atx	2003	42	26	8.1	↓
Biotronik Phylax AV	2000	49	41	0.0	↔

<u>CRT</u>

At 4 years

Mean value of all CRT-ICDs				80.2	
Boston Scientific Cognis	2008	76	1	97.5	
Biotronik Lumax 540 HF-T	2007	148	9	95.0	
Medtronic Concerto C 174	2006	52	23	93.4	
St. Jude Medical Promote RF 3213	2009	104	10	91.3	
Boston Scientific Contak 4 AVT M177 HE	2005	23	13	88.5	
Medtronic InSync III Marquis 7279	2005	47	28	57.1	
Boston Scientific Contak Renewal H135	2002	21	15	20.1	
Boston Scientific Contak Renewal 2 H155	2002	30	16	6.2	

At 5 years

Mean value of all CRT-ICDs				56.3	
Biotronik Lumax 540 HF-T	2007	148	9	89.2	↔
Medtronic InSync 7272	2002	25	9	77.8	↑
St. Jude Medical Promote RF 3213	2009	104	10	77.3	↔
Boston Scientific Contak Renewal 4 RF H230	2005	31	10	73.7	↑
Medtronic Concerto C 174	2006	52	23	72.4	↓
Medtronic InSync III Marquis 7279	2005	47	28	9.5	↔
Boston Scientific Contak Renewal H135	2002	21	15	0.0	↔
Boston Scientific Contak Renewal 2 H155	2002	30	16	0.0	↔

Supplemental table 3:

Longevity of devices grouped with a +/- similar CE/FDA approval year (at least 20 implants and 3 replacements) at the time points 4 and 5 years

Biotronik		year			4 years	5 years	mean replacement time (months)
Belos VRT/Lexos VRT/LexosA+/T	VVI	2003	94	58	89.8	59.8	60 ± 10
Lumos VR-T	VVI	2005	82	44	74.6	60.0	49 ± 21
Lumax VRT 300/340/540	VVI	2007	368	47	94.3	88.9	54 ± 22
Phylax AV	DDD	2000	49	41	5.7	0.0	33 ± 7
Lexos DRT/Tachos Atx/Tachos DR	DDD	2003	111	54	51.0	29.4	44 ± 18
Lumos DRT/Xelos DRT	DDD	2005	31	13	100.0	64.5	63 ± 6
Lumax DRT 300/340/540	DDD	2007	131	18	89.9	59.9	44 ± 18
Lumax HFT 340/540	CRT	2007	178	20	91.2	76.2	52 ± 17
Boston Scientific							
Ventak Prizm 1850/1860	VVI	2000	39	30	94.6	76.5	80 ± 20
Vitality 1870/T175/T177	VVI	2004	202	43	90.2	86.1	57 ± 20
Ventak Prizm 1851/1861/ 1900 AVT	DDD	2000	60	39	85.3	64.8	67 ± 20
Ventak Prizm HE 1853	DDD	2000	22	15	9.4	9.4	40 ± 8
Vitality 1871/A155/T165/T167	DDD	2004	154	59	90.1	73.8	59 ± 20
Contak CD 1823/Renewal 2 H155/H135	CRT	2002/3	62	35	22.1	8.3	42 ± 9
Contak Renewal 4 AVT M177/ H199 HE	CRT	2005/7	37	23	77.9	44.0	56 ± 12
Contak Renewal 4 H190/H195/H230/H235	CRT	2005/7	56	21	89.5	76.6	64 ± 11
Medtronic							
Jewel 7219/7220/7221/Microjewel 7221/7223	VVI	1994/98	28	24	92.6	54.0	71 ± 21
GEM 7227/7229/7231	VVI	1999/2000	148	106	96.4	89.1	92 ± 26
Marquis/Maximo/Entrust	VVI	2004/6	148	59	96.5	91.4	83 ± 21

Virtuoso D154VWC VR	VVI	2007	77	10	97.3	90.2	53 ± 13
Jewel AF 7250 H	DDD	1998	19	10	100.0	100.0	83 ± 13
GEM II DR 7271/7273/GEM III 7275/7276	DDD	1999/01	57	35	97.8	87.3	81 ± 19
Marquis/Maximo/Entrust/Intrinsic	DDD	2004/6	165	68	96.9	91.3	82 ± 20
Virtuoso D154AWG DR	DDD	2007	26	5	95.2	89.3	59 ± 18
InSync 7272	CRT	2002	25	9	85.6	77.8	61 ± 18
InSync Sentry/Marquis	CRT	2005	93	62	72.9	30.7	53 ± 15
Concerto/Consulta	CRT	2006/ 8	75	26	91.1	71.9	54 ± 20

<u>Sorin ELA</u>

Alto DR 614/624/Defender DR 612	DDD	2001/3 46	21		78.1	44.0	52 ± 16

<u>St. Jude Medical</u>

Profile 186	VVI	1999	20	12	0.0	0.0	35 ± 5
Atlas VR V-199	VVI	2001	72	36	84.2	73.2	63 ± 16
Atlas VR V-193	VVI	2003	103	17	94.3	91.5	67 ± 34
Atlas VR-V 199/193	VVI	2001/3	175	53	90.4	84.5	64 ± 23
Atlas II VR V-168	VVI	2006	105	4	97.5	97.5	53 ± 30
Current RF 1207/VR 1215	VVI	2007/9	89	4	96.0	96.0	40 ± 29
Ellipse CD 1277/1377	VVI	2010	77	1	n.a.	n.a.	7 ± 5
Fortify CD 1233	VVI	2010	138	7	n.a.	n.a.	24 ± 13
Atlas DR V-240	DDD	2001	59	41	73.3	19.4	52 ± 10
Atlas DR V-243	DDD	2003	62	21	94.7	94.7	73 ± 28
Atlas DR-V 240/243	DDD	2001/3	121	62	83.4	54.7	59 ± 20
Atlas II DR V-268	DDD	2006	59	15	91.2	75.6	54 ± 14
Current RF 2207/DR2215	DDD	2007/9	51	3	93.3	93.3	13 ± 13
Ellipse CD 2277/2377	DDD	2010	64	3	n.a.	n.a.	6 ± 4
Fortify CD2233/CD2259	DDD	2010	94	2	97.9	n.a.	1 ± 1
Atlas HF V-341/Epic V-339	CRT	2004	119	47	84.6	61.5	56 ± 13
Atlas HF V-366/V-367	CRT	2006/7	144	56	66.5	37.8	47 ± 11

Promote 3215/Quadra/RF3213	CRT	2009	106	10	91.4	77.4	39 ± 23
Unify CD 3235/3251/3235/3261/3361	CRT	2010/2	156	3	n.a.	n.a.	19 ± 10
