# Supplementary figures and images for: Longevity of implantable cardioverter defibrillators: a comparison among manufacturers and over time
Source: Europace. 2015 Nov 25;18(5):710–7. doi: 10.1093/europace/euv296 (PMC4880113; doi:10.1093/europace/euv296)

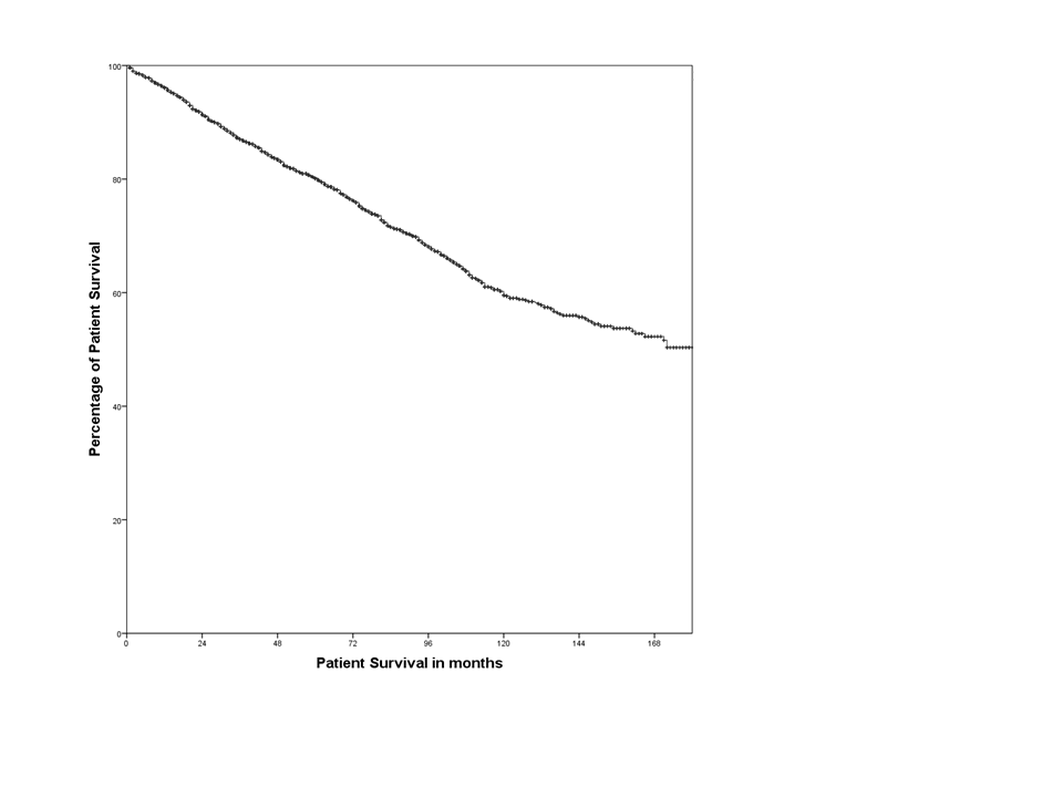

Supplement: Supplementary Data [file euv296_supplementary_data.zip › euv296supp_fig1.tif]

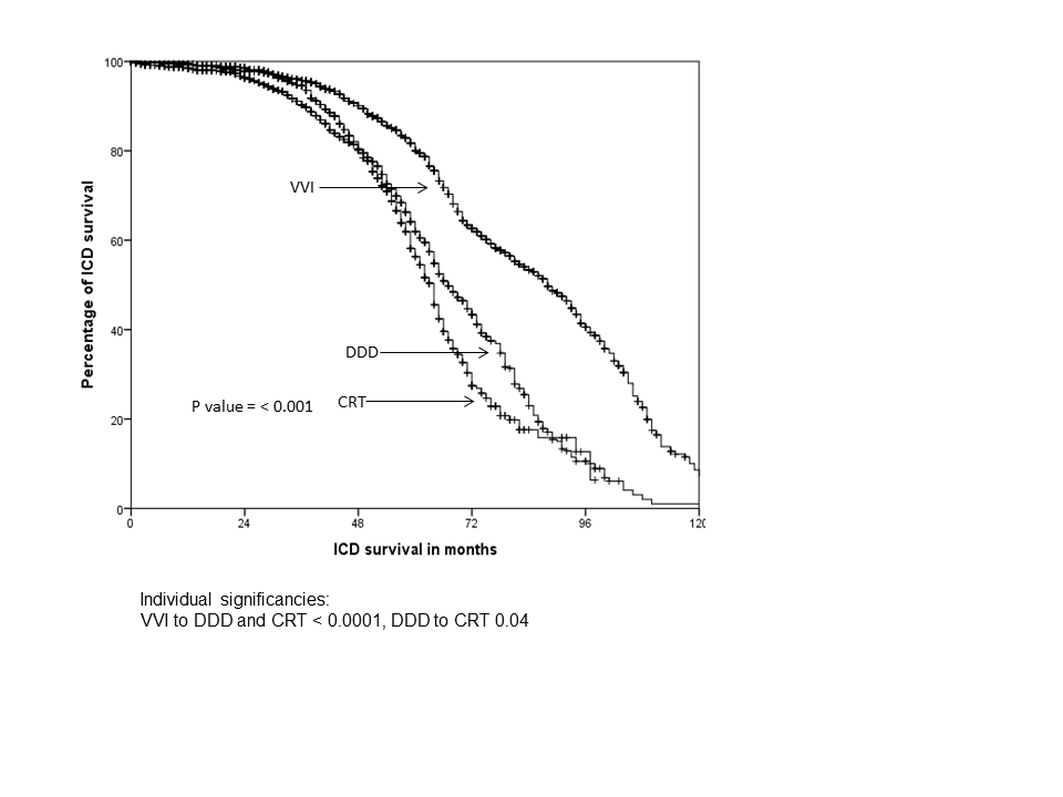

Supplement: Supplementary Data [file euv296_supplementary_data.zip › euv296supp_fig3.tif]

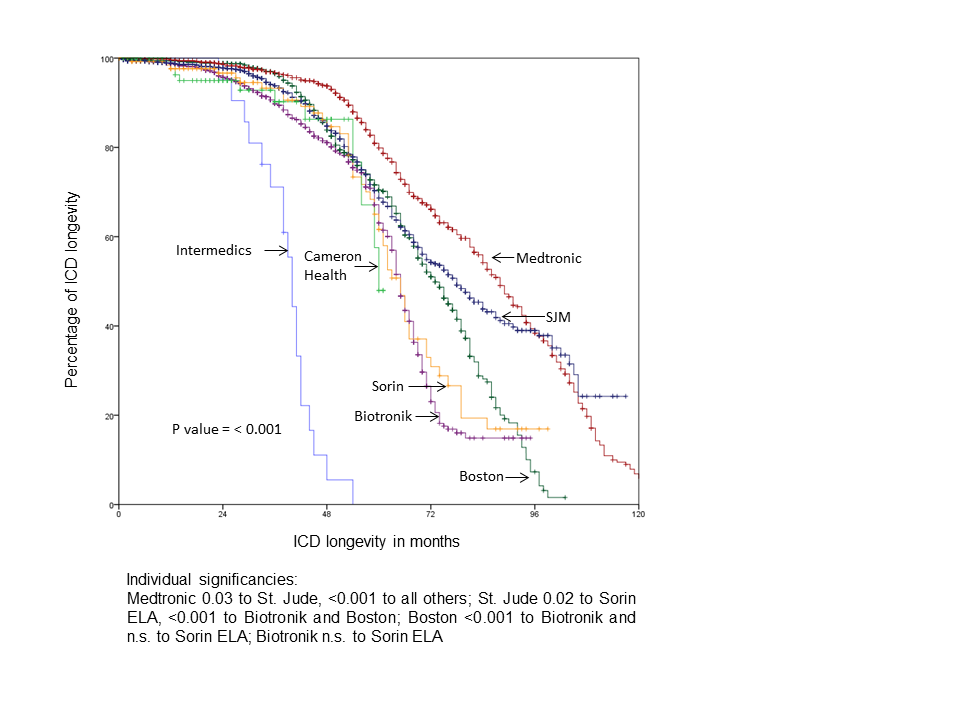

Supplement: Supplementary Data [file euv296_supplementary_data.zip › euv296supp_fig2.tif]
